# Obesity Aggravates Acute Pancreatitis via Damaging Intestinal Mucosal Barrier and Changing Microbiota Composition in Rats

**DOI:** 10.1038/s41598-018-36266-7

**Published:** 2019-01-11

**Authors:** Cheng Ye, Ling Liu, Xiao Ma, Huan Tong, Jinhang Gao, Yang Tai, Libin Huang, Chengwei Tang, Rui Wang

**Affiliations:** 1 Department of Gastroenterology, West China Hospital, Sichuan University, Chengdu, China; 2 Division of Peptides Related with Human Diseases, State Key Laboratory of Biotherapy, West China Hospital, Sichuan University, Chengdu, China

## Abstract

Obesity may aggravate acute pancreatitis (AP) through damaging the intestinal mucosal barrier (IMB). The underlying mechanism remains unclear. This study was aimed to provide further data to clarify the mechanism. 48 rats were divided into 4 groups: 1) normal control (NC), chow-fed rats with sham operation, 2) no-obese rats with AP (NAP), chow-fed rats with taurocholate infusion, 3) obese control (OC), high-fat diet (HFD)-fed rats with sham operation, and 4) obese rats with AP (OAP), HFD-fed rats with taurocholate infusion. Pancreatic pathologic score (11.39 ± 1.76 *vs*. 14.11 ± 1.05, *p* = 0.005), intestinal permeability to FD4 (0.91 ± 0.25 μg/ml *vs*. 7.06 ± 3.67 μg/ml, *p* < 0.001), serum leptin (10.25 ± 5.59 ng/ml *vs*. 79.73 ± 38.44 ng/ml, *p* < 0.001) and ileal apoptosis (2.05 ± 0.73% *vs*. 4.53 ± 2.28%, *p* = 0.006) were significantly higher in OAP than in NAP group. The intestinal bacterial richness (Chao 1 and OTUs) was significantly lower in OAP than in NAP rats. The higher abundance of Proteobacteria and reduced proportions of intestinal Actinobacteria, *Allobaculum and Barnesiella* were detected in OAP group. Obesity may result in decreased intestinal leptin/ObR-b binding, distinct phylogenetic clusters of ileal bacterial communities, increased intestinal inflammatory injury and the insufficient intestinal epithelial cells proliferation during AP attack. Pancreatic injury was aggravated due to obesity associated dysfunction of IMB.

## Introduction

Severe acute pancreatitis (SAP) with the characters of persistent organ failure or infectious local complications has been taken as one of the most terrible celiac disorders, with a mortality rate as high as 10–30%^1^. Dysfunction of the intestinal mucosal barrier (IMB) plays an essential role in the development of SAP^2,3^. Barrier dysfunction, usually due to dysregulated tight junctions (TJs) and/or aberrant apoptosis or proliferation of the intestinal epithelial cells (IECs), can lead to increased barrier permeability and invasion of luminal antigens and bacteria into the lamina propria, triggering immune cell-mediated mucosal inflammation^2,4^. Elevated intestinal permeability (IP) is a main cause of bacterial and endotoxin translocation in the progression of SAP^2,5^. In addition, obesity, which is considered to induce a chronic inflammatory state per se^6^, has been considered an independent risk factor for SAP. The occurrences of systemic and local complications may increase to 1.68–2.8-fold in obese patients^7,8^. Obesity has been reported to damage the IMB due to the down-regulation of TJs^9^. However, the status of IMB under obesity during acute pancreatitis (AP) is unknown. The underlying mechanism remains unclear.

Leptin, a 16-kilodalton product of the *ob* gene, is considered pivotal in the energy homeostasis in obesity^10^. It is not only released from adipose tissue but also synthesized from intestinal mucosa^11^. Apart from metabolic regulation, recent studies have shown that the binding of leptin to long form leptin receptor ObR-b may induce proliferation and reduce apoptosis of IECs during intestinal ischaemia/reperfusion^12,13^. However, there is no data on intestinal leptin level in the obese one with SAP. The association between intestinal leptin and IEC repair is largely unknown.

A healthy gut microbial ecosystem is thought to be characterized by high bacterial richness and diversity, which are presumed to reflect the stability and resilience of the IMB^14^. Accumulating reports have shown the relationship between intestinal dysbiosis and progression of digestive diseases. Patients with inflammatory bowel disease have a higher proportion of Proteobacteria^15^ and the abundance of Actinobacteria in peptic ulcer released patients is increased^16^. The development and progression of fatty liver appears to be influenced by the composition of the microbiota^17^. As for the condition of acute pancreatitis, alteration of microbiota composition has been reported in the development of SAP^18^. However, whether obesity affects bacterial richness and diversity or microbiota composition during AP is also an interesting issue to answer.

This study was aimed to provide further data on intestinal permeability (IP), IEC repair, intestinal leptin/ObR-b binding and microbiota during AP attack to clarify that obesity may aggravate pancreatic injury through IMB dysfunction.

## Results

### Obesity aggravated acute pancreatitis

**Figure 1:**
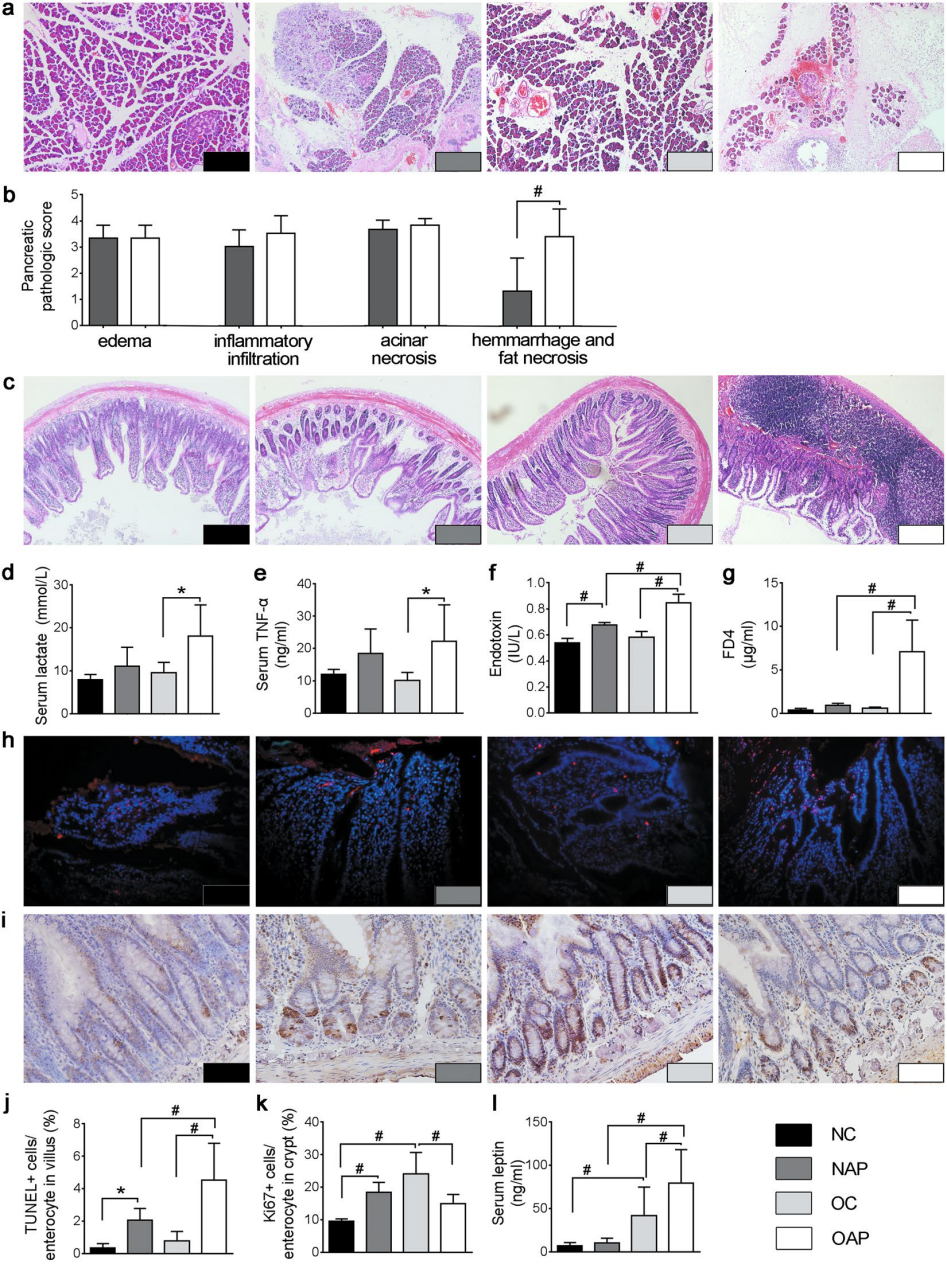
Histopathological changes of the pancreas and ileum with quantitative comparisons. (**a**,**b**) Histopathological changes of the pancreas (H&E staining, ×100 magnifications). (**c**) Histopathological changes of the ileum (H&E staining, ×100 magnifications). (**d**) Serum lactate level. (**e**) Serum level of TNF-α. (**f**) Portal vein endotoxin level. (**g**) FD4 absorption. (**h**,**j**) Apoptotic bodies (red) in the intestinal epithelium (TUNEL, ×400 magnifications). (**i**,**k**) Proliferation of ileal epithelium (Immunohistochemical stain of Ki67, ×400 magnifications). (**l**) Serum levels of leptin. Each value is the mean ± SD from 8 animals in each group, and duplicate measurements were made. *p < 0.05, ^#^*p* < 0.01.

A high-fat diet (HFD) for 6 months led to an increased body weight, Lee’s index and concentrations of cholesterol and triglycerides (Supplementary Table 1). Animal groupings: normal weight rats with sham operation (NC), normal weight rats with AP induction (NAP), obese rats with sham operation (OC), and obese rats with AP induction (OAP). Serum levels of amylase in two AP groups were significantly increased after retrograde infusion of Na-taurocholate (NC 1486.53 ± 403.56 U/L *vs*. NAP 2819.90 ± 476.46 U/L, *p* = 0.006; OC 1460.68 ± 565.37 U/L *vs*. OAP 2896.84 ± 153.45 U/L, *p* = 0.001). Also, Schmidt’s acute pancreatic damage scores were much higher in AP groups than in the corresponding control groups (NC 3.32 ± 1.31 *vs*. NAP 11.39 ± 1.76, *p* < 0.001; OC 3.94 ± 2.10 *vs*. OAP 14.11 ± 1.05, *p* < 0.001). Moreover, the pathologic score in the OAP group was significantly higher than that of the NAP group, *p* = 0.005 (Fig. 1a,b). Serum lactate and tumour necrosis factor (TNF)-α were significantly increased only in OAP rather than in NAP, compared with their corresponding controls (Fig. 1d,e).

### Obesity deteriorated intestinal permeability of rats with AP

Rats with AP (NAP) showed higher serum endotoxin level (0.65 ± 0.06 IU/L) than control ones (NC 0.54 ± 0.04 IU/L), *p* = 0.009. However, the serum endotoxin level in obese rats with AP (OAP 0.85 ± 0.07 IU/L) was not only higher than that in its corresponding control group (OC 0.57 ± 0.03 IU/L), *p* < 0.001, but was also much higher than that in NAP (0.85 ± 0.07 IU/L *vs*. 0.65 ± 0.06 IU/L, *p* < 0.001; Fig. 1f). Interestingly, the NAP group did not show significantly higher IP to a 4-kDa macromolecular fluorescent probe (FD4) when compared to the NC group, *p* > 0.05. The OAP group not only did it when compared with the OC group, but also showed significantly higher FD4 absorption than the NAP group, *p* < 0.001 (Fig. 1g). Obesity was associated with elevation of endotoxin translocation and FD4 absorption in AP rats.

### Obesity aggravated intestinal inflammatory injury during AP

Obvious inflammatory injuries were shown in the intestines of obese rats with AP (Fig. 1c). The histopathological scores of the intestines in OAP were significantly higher than those in NAP (5.39 ± 1.24 *vs*. 4.45 ± 1.04, *p* = 0.038). Furthermore, elevation of pathologic scores in the OAP group mainly referred to leukocyte infiltration (*p* = 0.002) rather than mucosal injury (*p* = 0.546).

### Obesity led to more apoptosis but less proliferation of IECs during AP

AP induced significant apoptosis of IECs in both NAP and OAP when compared with their corresponding controls. The apoptosis index of IECs in the OAP group was significantly higher than that in NAP (Fig. 1h,j). IEC proliferation with Ki67 expression in the ileal crypt was increased in the NAP group (18.37 ± 3.12%) when compared with the NC group (9.52 ± 0.79%, *p* = 0.008). In contrast, with the same induction of AP, obesity greatly inhibited the IEC proliferation. Ki67 expression in ileal crypt of the OAP group (14.89 ± 2.89%) was significantly lower than that in the OC group (24.03 ± 6.64%), *p* = 0.003 (Fig. 1i,k). Obviously, obesity led to more apoptosis but less proliferation of IECs during AP.

### Obesity and AP did not affect the expression of TJ proteins between ileal IECs

There was no morphological change of the paracellular gaps between ileal IECs among the four groups (Supplementary Fig. 1a). Expressions of TJ proteins (occludin and claudin-1) did not significantly differ among the four groups by Western blot (WB) analysis or RT-qPCR (*p* > 0.05; Supplementary Fig. 1b,c).

### Obesity resulted in imbalanced expression between leptin and intestinal ObR-b during AP

Compared with the NC group, the serum level of leptin of the OC group was increased, *p* = 0.004. After induction of AP, the serum level of leptin of the OAP group was not only higher than that of the OC group, but also much higher than that of the NAP group. Only on the basis of obesity, AP may promote leptin releasing from adipose tissue (Fig. 1l).

**Figure 2:**
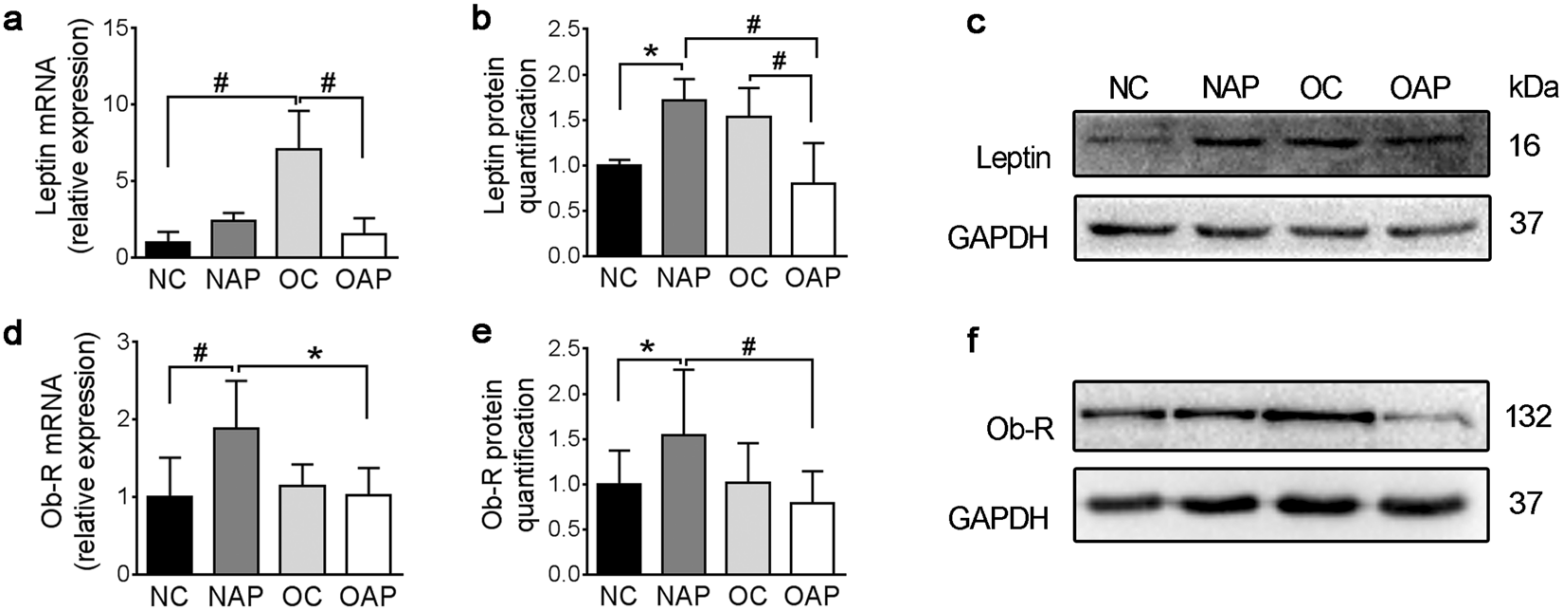
Quantitation of ileal leptin and ObR. (**a**) mRNA expression of ileal leptin. (**b**) Protein quantification of ileal leptin. (**c**) Western blot analysis of ileal leptin. (**d**) mRNA expression of ileal ObR. (**e**) Protein quantification of ileal ObR. (**f**) Western blot analysis of ileal ObR. Each set of samples run in parallel. Each value is the mean ± SD from 8 animals in each group and duplicate measurements were made. *p < 0.05, ^#^*p* < 0.01. Full-length blots were presented in Supplementary Fig. 2.

Similarly, compared with the NC group, the ileal leptin of OC group was significantly increased. However, after induction of AP, the ileal leptin of OAP was decreased by nearly 50% when compared with the OC group (Fig. 2a–c). The ileal leptin protein level was significantly correlated with IEC proliferation (r^2^ = 0.411, *p* = 0.007) instead of apoptosis (r^2^ = 0.224, *p* = 0.064).

Expression of ileal ObR-b was increased with the induction of AP but not with obesity (Fig. 2d–f). Interestingly, ileal ObR-b expression in obese rats were not stimulated with the induction of AP (RT-qPCR: NAP 1.88 ± 0.62 *vs*. OAP 1.02 ± 0.356 relative expression, *p* = 0.012; WB: NAP 1.54 ± 0.72 *vs*. OAP 0.79 ± 0.35 relative quantification, *p* = 0.006; Fig. 2d–f).

### Obesity changed ileal microbial diversity and composition of rats with AP

**Figure 3:**
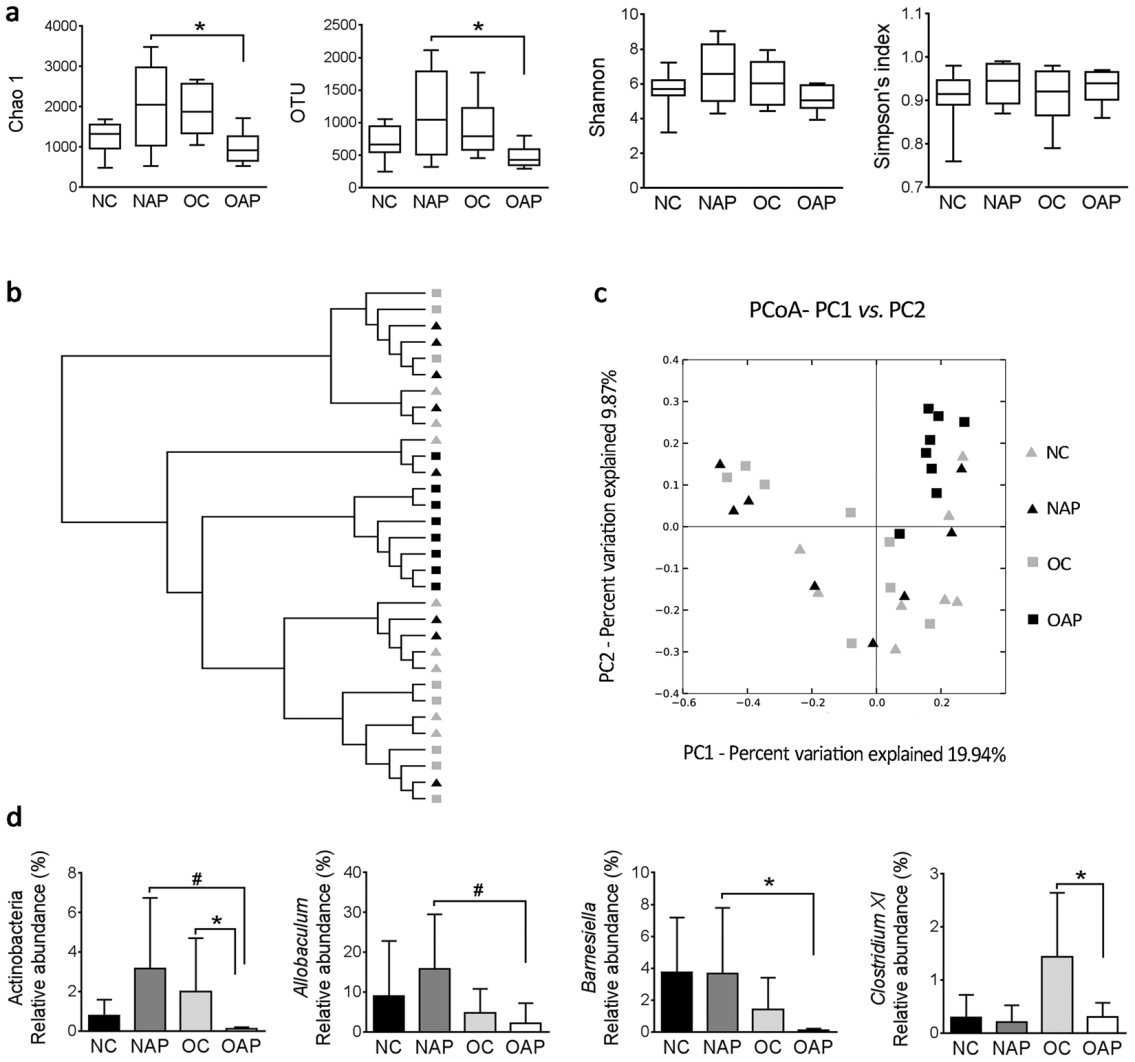
Alpha diversity, beta diversity and microbiome composition of the bacterial communities. (**a**) The Chao 1, OTUs, and Shannon and Simpson indexes were used to estimate the richness and diversity of the intestinal microbiota. (**b**) Unweighted Pair Group Method with Arithmetic mean (UPGMA) tree based on unweighted Unifrac distance matrix. (**c**) PCoA, based on unweighted Unifrac distance, was used to reveal the structural segregation of bacterial communities among the four groups. (**d**) Abundance of Actinobacteria and proportions (genus level) of *Allobaculum*, *Barnesiella* and *Clostridium XI* among the four groups. *p < 0.05, ^#^*p* < 0.01. n = 8 in each group and duplicate measurements were made.

The alpha diversity values (Chao 1 richness, OTU, Shannon’s diversity and Simpson index) of the four groups were compared (Fig. 3a). There were no significant differences in alpha diversity between NC and OC groups, *p* > 0.05. The levels of Chao 1 and OTUs in OAP were significantly lower than that in NAP, Chao 1 *p* = 0.019; OTU *p* = 0.026 (Fig. 3a). With regarding of beta diversity, principal coordinate analysis (PCoA), based on unweighted Unifrac distance, was used to reveal the structural segregation of rat ileal bacterial communities among the four groups (Fig. 3b,c). There was no significant difference in beta diversity of gut microbiota between groups of NC and OC, *p* > 0.05. However, a distinct gut microbiota was correlated with either OAP or NAP (Bray-Curtis, NAP vs. OAP r^2^ = 0.18, *p* = 0.028).

With microbiome composition analysis, the most abundant phyla in all the groups included Proteobacteria, Firmicutes and Bacteroidetes. The abundance of Proteobacteria in OAP (59.59%) was higher than that in the OC group (23.56%), *p* = 0.013. With induction of AP, obesity significantly reduced intestinal Actinobacteria proportion, NAP 3.15% *vs*. OAP 0.11%, *p* = 0.007 (Fig. 3d). And the abundance of Actinobacteria in obese rats with AP was lower than that in obese controls (OC 1.99% *vs*. OAP 0.11%, *p* = 0.014, Fig. 3d). At the genus level, similar data were also shown in the proportions of *Allobaculum* (NAP 15.85% *vs*. OAP 1.85%, *p* = 0.008, Fig. 3d) and *Barnesiella* (NAP 3.66% *vs*. OAP 0.06%, *p* = 0.018, Fig. 3d). The proportion of *Clostridium XI* was not changed in normal weight rats after induction of AP but was significantly decreased in obese rats with AP when compared with its control group (Fig. 3d). Moreover, there was a positive correlation of *Clostridium XI* abundance with IEC proliferation (r^2^ = 0.730, *p* < 0.001).

## Discussion

**Figure 4:**
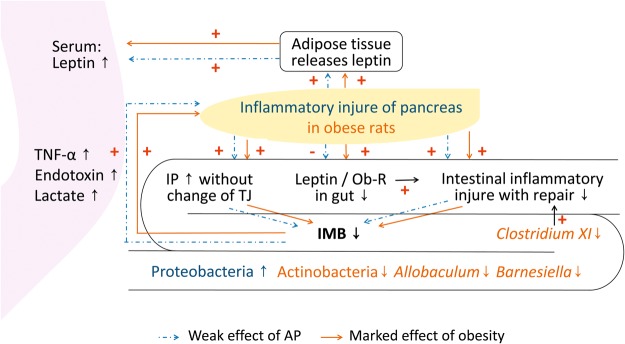
Schema of aggravated pancreatic injury due to obesity IP: intestinal permeability, TJ: tight junction, Ob-R: leptin receptor, IMB: intestinal mucosal barrier.

Several epidemiological studies have suggested that obesity^7^ or increased intra-abdominal fat was associated with the development of SAP^17^. This study provided further data to clarify the dysfunction of the IMB in aggravation of pancreatic injury by obesity (Fig. 4). Either AP or obesity was involved in several pathways at the same time. However, obesity presented marked effects compared with AP.

The sealing property of intestinal mucosa requires an anatomically intact epithelium as well as efficient apical cell junctional complexes. Many insults may cause mucosal erosion or necrosis, imbalance of epithelial apoptosis and proliferation as well as disorganization of TJ proteins^5,9,18,19^. Different from previous reports, in which down-regulation of intestinal TJs was associated with AP^2,5^ and obesity^20^, the expressions of TJ proteins between ileal IECs were not affected in this study. The IP was increased in obese rats during AP and was characterized by more apoptosis but less proliferation of IECs. The insufficient IEC proliferation would be hardly enough to make up for the increased apoptosis during AP. Instead of obvious mucosal erosion or ulcer, the fragile IMB may be in the earlier stage of dysfunction of IMB in this animal model. Even though, the pancreatic inflammation was still aggravated through the translocation of endotoxin, lactate and TNF-α and activation of intestinal innate immunity. Excessive IEC apoptosis may be the major cause of increased mucosal inflammation at the early stage of AP in the obese rats.

IEC apoptosis and proliferation are regulated by several factors. Other than produced by adipose tissue, intestinal leptin may be one of these factors with the ability of accelerating proliferation and reducing apoptosis of IECs^13,21^. AP promoted leptin releasing from adipose tissue but reduced the intestinal leptin in the obese rats in this study. Among the 6 types of leptin receptors, long-form ObR-b is the only isoform with complete transmembrane and intracellular domains^22^. ObR-b is also present throughout the intestinal epithelium, both in apical and basolateral membranes of IECs^23,24^, suggesting that leptin and ObR-b may participate in the physiological regulation of IMB, perhaps by a paracrine pathway. Previous studies also suggested that leptin/ObR-b and inflammatory mediators were closely affected by each other^25–27^. Leptin was speculated to display a dual nature: as a mediator of inflammation, and as a growth factor for the intestine^28^. Therefore, although the expression of ObR-b was not markedly changed, less intestinal leptin/ObR-b binding in the obese rats with AP might affect the function of IMB through the deterioration of IEC proliferation. Moreover, dramatic release of circulating leptin by adipose tissue did not enhance intestinal leptin or stimulate ObR expression, suggesting a paracrine action of intestinal leptin in this study.

In addition, previous reports have shown that leptin may regulate the composition of gut microbiota. Either leptin-deficient (*ob/ob*) or leptin receptor-deficient (*db/db*) mice had greatly altered gut microbial communities compared with wild-type littermates^29,30^. Consistent with these observations, less intestinal leptin/ObR-b binding was associated with the change of ileal microbiota composition in the obese rats with AP in this study. *Clostridium* has previously been reported to have anti-inflammatory effect and to promote IEC proliferation with its butyrate producing capability^31,32^. The greatly decreased proportion of *Clostridium XI* in obese rats with AP might be related to the intestinal inflammatory injury and the lack of repair. These results were only observational and speculative. The exact impacts of leptin and ObR-b on IMB and gut microbiota on obesity with AP need further experiments.

Faecal microbiome analyses have suggested bacterial richness to be a major marker for gut health^14,33^. The intestinal bacterial richness (Chao 1 and OTUs) was significantly lower in obese rats than no-obese rats under the condition of AP in this study. It is in line with previous reports of a decreased diversity of the faecal microbiome with obesity^34,35^. The ileal bacterial communities of obese rats with AP presented the distinct phylogenetic clusters compared with no-obese rats with AP. The higher abundance of Proteobacteria and reduced proportions of intestinal Actinobacteria, *Allobaculum and Barnesiella* were detected in the present study. It is unclear what action on IMB would be caused with such microbial dysbiosis. Recent reports have shown that higher Proteobacteria proportion was observed in patients who developed necrotizing enterocolitis^36^. Ileal abundances of Actinobacteria, *Allobaculum*, and *Barnesiella* were shown to be associated with reduction of intestinal ulceration^16^, with short chain fatty acids producing capability^37^ and to protect patients from Clostridium difficile infection^38^. Therefore, the microbial dysbiosis happened in the obese rats with AP in this study might deteriorate the dysfunction of IMB and subsequent sepsis^39^. Faecal microbiota analysis is usually used in many studies. However, faecal sample for gut microbiome analysis might contain bacteria from unspecified gastrointestinal tract compartments^40^. This study focused on the interaction between the ileal microbiota and IMB. The ileal content, easier to collect in animal research than in human study, was analysed. It may be more specified than faecal samples (Supplementary data) which contain the colonic microbiota.

In conclusion, obesity may result in the decreased intestinal leptin/ObR-b binding, lower bacterial richness, distinct phylogenetic clusters of ileal bacterial communities, increased intestinal inflammatory injury and the insufficient IEC proliferation during AP attack. All of these interacted with each other and aggravated pancreatic injury through the dysfunction of IMB, even at its early stage.

## Methods

### Animals

Animal protocols were approved by the Animal Use and Care Committee of Sichuan University and were conducted according to the regulations set by Sichuan University. Adult male *Sprague-Dawley* (SD) rats (180–200 g, n = 48) were purchased of the same batch from Sichuan University Laboratory Animal Center. All rats were raised in separate cages (1 cage for 1 rat) with the same humidity, temperature, a 12:12 h light/dark cycle and had free access to food and water *et al*. All of the rats were fed with conventional chow die during the adaption.

### Inductions of obesity, AP and grouping

Obese rats were induced by a high-fat diet (HFD) containing 54 kcal % fat^41,42^. The rats in control group were fed with conventional chow diet. All the animals had free access to food and tap water for 6 months. Rats were fasted for 12 h before operation. Anaesthesia was achieved with 1% pentobarbital sodium (Merck KGaA, Darmstadt, Germany). A midline laparotomy was performed, and the first loop of the duodenum was identified. AP was induced in a well-established AP model, which can be used to induce biliary pancreatitis^43^, by retrograde infusion of 5% Na-taurocholate (Sigma, St. Louis, MO, USA) to the bile-pancreatic duct (1 ml/kg), at a speed of 100 μl/min using an infusion pump, as previously described^44^. After operation, animals were given free access to 5% glucose-saline solution. Rats undergoing catheter insertion into the bile-pancreatic duct without any fluid infusion served as a sham operation control.

A total of 48 rats were divided into 4 groups (12 rats in each group): 1) normal control (NC), chow-fed rats with sham operation, 2) no-obese rats with AP (NAP), chow-fed rats with taurocholate infusion, 3) obese control (OC), HFD-fed rats with sham operation, and 4) obese rats with AP (OAP), HFD-fed rats with taurocholate infusion. Those rats were sacrificed under anaesthesia 24 h after the pancreatic duct insertion.

### Measurements of serum levels of lipids and amylase

Serum was collected and tested for cholesterol, triglycerides and amylase levels by an Olympus AU2700 analyser (Olympus, Tokyo, Japan) based on the recommendations of the International Federation of Clinical Chemistry.

### Serum lactate concentration

Serum lactate was quantified from cardiac puncturation using a Lactic Acid LD kit (KeyGEN BioTECH, Nanjing, China). Plates were read using the microplate reader under the wavelengths of 530 nm.

### Histopathological study of pancreatitis and intestinal mucosal damage

Pancreatic and terminal ileal tissues fixed in 4% neutral buffered paraformaldehyde were embedded in paraffin, sectioned, and then stained with haematoxylin and eosin (H&E). The morphological changes were examined under a microscope (CX41, Olympus, Tokyo, Japan) equipped with a digital camera (DP72, Olympus, Tokyo, Japan). The severity of pancreatitis and intestinal damage were evaluated, as reported previously^45,46^.

### Endotoxin and FITC-dextran assay for evaluation of IP

Concentration of serum endotoxin from the portal vein was quantified with a Chromogenic End-point TAL kit according to the manufacturers’ instructions (Chinese Horseshoe Crab Manufactory, Xiamen, China). Plates were read using the microplate reader under the wavelengths of 545 nm. Twenty-four hours after bile-pancreatic duct infusion, a midline laparotomy was performed under anaesthesia. Then, fluorescein isothiocyanate (FITC) dextran of 4 kDa (FD4, Sigma, St. Louis, MO, USA) dissolved in PBS (40 mg/kg) was injected into the surgically exposed jejunal segments using a micro-syringe. One hour after injection, blood suction was performed by cardiac puncture and centrifuged (3000 × g, 10 min, 4 °C). The fluorescence intensity (λex/λem 485/525 nm) was detected using a spectrofluorimeter (Bio Tek, VT, USA). Serum concentrations of FITC-dextran were subsequently measured against a standard curve.

### Measurements of serum cytokine and leptin

The concentrations of serum TNF-α and leptin from cardiac puncturation were quantified with enzyme-linked immunosorbent assay (ELISA) kits according to the manufacturers’ instructions (USCN Life Sciences Inc., Wuhan, China). Plates were read using the microplate reader (Bio Tek, VT, USA) under the wavelengths of 450 nm.

### Immunohistochemical study for proliferation of IECs

Sections of terminal ileal tissues were deparaffinized in xylene and serial ethanol dilutions. Antigen retrieval was performed by heating the sections in 10 mM sodium citrate buffer. Sections were blocked and incubated with primer antibody to Ki-67 (1:1000, Thermo Fisher Scientific, MA, USA) overnight at 4 °C and developed with biotinylated secondary antibodies, and then incubated with streptavidin-biotin-complex. The sections were then stained with a solution of 3,3-diaminobenzidine tetrahydrochloride and counterstained with haematoxylin. The number of crypt enterocytes which expressed Ki-67 was calculated as the percentage of total crypt enterocytes.

### Detection of ileal enterocyte apoptosis

DNA fragmentation on ileal tissue (paraffin session) was assayed by terminal deoxynucleotidyl transferase-mediated dUTP nick-end labelling (TUNEL) assay (Roche, Mannheim, Germany). The number of TUNEL-positive enterocytes was calculated as the percentage of total surface enterocytes.

### Measurement of intestinal occludin, claudin-1, leptin and ObR-b

Twenty-four hours after AP establishment, ileal tissues were stored at −80 °C until protein and RNA analysis. Ileal expressions of occludin, claudin-1, leptin and ObR-b were measured by Western blot, as previously described^47^. Samples were probed with primary antibodies against occludin (1:125, Invitrogen, CA, USA), claudin-1 (1:100, Abcam, Cambridge, UK), leptin (1:1000, Abcam, Cambridge, UK) and leptin receptor (ObR-b) (1:1000, Abcam, Cambridge, UK) overnight at 4 °C. Protein expressions were determined using Bio-Rad image lab software 5.1 (Bio-Rad Laboratories, Hercules, CA, USA). The housekeeping protein GAPDH was applied as an internal control.

Total RNA was extracted from rat ileal tissues by TRIzol reagent (Invitrogen, CA, USA). One microgram of total RNA was used for first-strand complementary DNA synthesis employing the PrimeScript RT reagent kit (TaKaRa Bio Inc., Japan). Primer sequences were given in Supplementary Table 2. Then, iQ SYBR Green Supermix (Bio-Rad Laboratories, Hercules, CA, USA) was applied as a fluorescent dye to detect the presence of double-stranded DNA. The mRNA values for each gene were normalized to GAPDH mRNA using −2^ΔΔCt^ method.

### Ileal contents sampling, DNA extraction and sequencing

Briefly, 24 h after AP induction, ileal segments were opened. The ileal contents were collected and immediately stored at −80 °C for DNA extraction (n = 8 in each group). DNA extraction was proceeded using a DNA isolation kit (MoBio Laboratories Inc., Carlsbad, USA) following the manufacturers’ instructions. PCR amplification of the V4-V5 hypervariable region of the 16S rRNA gene was done using universal primers 515F (5′-GTGCCAGCMGCCGCGGTAA-3′) and 806R (5′-GGACTACVSGGGTATCTAAT-3′). The PCR mixture (50 μl) contained 0.5 unit Ex Taq DNA polymerase, 10 μl 1× Ex Taq buffer, 8 μl dNTP mix (TaKaRa Bio Inc., Japan), 2 μl of each primer (10 mM) and 10–100 ng template DNA. The PCR amplification program included initial denaturation at 95 °C for 3 min, followed by 30 cycles of 94 °C for 30 s, 56 °C for 60 s, 72 °C for 60 s; and a final extension at 72 °C for 10 min. Triplicate PCR reactions were performed per sample and pooled for purification using the Gel Extraction kit (Omega bio-tek, GA, USA). Equal molar of PCR product from each sample was pooled together. The sequencing library was prepared using Truseq DNA PCR-Free Library Preparation Kits, and sequenced at Illumina Miseq platform with 2 × 250 bp V2 kits at the Environmental Genome Platform of Chengdu Institute of Biology.

Raw sequences were sorted based on unique sample tags, trimmed for sequence quality, and denoised using the Quantitative Insights into Microbial Ecology (QIIME) pipeline^48^. Chimera sequences were removed using the Uchime algorithm^49^. Each sample was rarefied to 8950 reads (i.e., the fewest number of sequences in a single sample). The sequences were clustered by the complete-linkage clustering method incorporated in QIIME. Operational taxonomic units (OTUs) were classified using the 16 S rRNA gene with a 97% identity threshold. Chao1 index and Shannon and Simpson indexes were calculated using the Ribosomal Database Project (RDP) pipeline with a 97% sequence identity (http://pyro.cme.msu.edu/). PCoA, based on unweighted Unifrac distance, was conducted to perceive the differences in bacterial community structure among the groups. The Bray-Curtis dissimilarity matrix performed with permutational multivariate analysis of variance (PERMANOVA) was used to compare community similarity based on OTU abundance.

### Statistical analysis

All variables were measured in duplicate. And all data are expressed as the mean ± SD and analysed by SPSS 20.0 software (SPSS, Chicago, IL, USA). Differences in the physiological and biochemical data and alpha diversity measures among different groups were tested using one-way analysis of variance (ANOVA) followed by Tukey’s multiple comparison test. The Kruskal–Wallis test (adjusted *p* value) was used to analyse data of microbiota composition. Correlations between ileal leptin level and IEC proliferation, and between the proportion of *Clostridium XI* and IEC proliferation were analysed using Pearson correlation coefficient. A value of *p* < 0.05 was considered significant.

## Electronic supplementary material

Supplementary information

## Data Availability

All data generated or analysed during this study are included in this published article (and its Supplementary Information Files). The original 16S rRNA data were available at the European Nucleotide Archive by accession no. PRJEB26873 (http://www.ebi.ac.uk/ena/data/view/PRJEB26873).

## References

[1] Steinberg W, Tenner S (1994). Acute pancreatitis. The New England journal of medicine.

[2] Sonika U (2016). Mechanism of Increased Intestinal Permeability in Acute Pancreatitis: Alteration in Tight Junction Proteins. Journal of clinical gastroenterology.

[3] Andersson R (1998). Effect of a platelet-activating factor antagonist on pancreatitis-associated gut barrier dysfunction in rats. Pancreas.

[4] He L (2018). Gut Epithelial Vitamin D Receptor Regulates Microbiota-Dependent Mucosal Inflammation by Suppressing Intestinal Epithelial Cell Apoptosis. Endocrinology.

[5] Merilainen S (2012). Intestinal bacterial translocation and tight junction structure in acute porcine pancreatitis. Hepato-gastroenterology.

[6] Frossard J-L, Lescuyer P, Pastor CM (2009). Experimental evidence of obesity as a risk factor for severeacute pancreatitis. World Journal of Gastroenterology.

[7] Chen SM, Xiong GS, Wu SM (2012). Is obesity an indicator of complications and mortality in acute pancreatitis? An updated meta-analysis. Journal of digestive diseases.

[8] Krishna SG (2015). Morbid Obesity Is Associated With Adverse Clinical Outcomes in Acute Pancreatitis: A Propensity-Matched Study. The American journal of gastroenterology.

[9] Brun P (2007). Increased intestinal permeability in obese mice: new evidence in the pathogenesis of nonalcoholic steatohepatitis. American journal of physiology. Gastrointestinal and liver physiology.

[10] Zhang Y (1994). Positional cloning of the mouse obese gene and its human homologue. Nature.

[11] Hansen GH, Niels-Christiansen LL, Danielsen EM (2008). Leptin and the obesity receptor (OB-R) in the small intestine and colon: a colocalization study. The journal of histochemistry and cytochemistry: official journal of the Histochemistry. Society.

[12] Fenton JI, Hursting SD, Perkins SN, Hord NG (2006). Interleukin-6 production induced by leptin treatment promotes cell proliferation in an Apc (Min/+) colon epithelial cell line. Carcinogenesis.

[13] Deng ZH (2012). Leptin relieves intestinal ischemia/reperfusion injury by promoting ERK1/2 phosphorylation and the NO signaling pathway. The journal of trauma and acute care surgery.

[14] Lozupone CA, Stombaugh JI, Gordon JI, Jansson JK, Knight R (2012). Diversity, stability and resilience of the human gut microbiota. Nature.

[15] Bradley PH, Pollard KS (2017). Proteobacteria explain significant functional variability in the human gut microbiome. Microbiome.

[16] Wallace, J. L. *et al*. Proton pump inhibitors exacerbate NSAID-induced small intestinal injury by inducing dysbiosis. *Gastroenterology* **141**, 1314–1322, 1322 e1311-1315, 10.1053/j.gastro.2011.06.075 (2011). 10.1053/j.gastro.2011.06.075 21745447

[17] Hall TC (2015). Is Abdominal Fat Distribution Measured by Axial CT Imaging an Indicator of Complications and Mortality in Acute Pancreatitis?. Journal of gastrointestinal surgery: official journal of the Society for Surgery of the Alimentary Tract.

[18] Yasuda T (2006). Breakdown of intestinal mucosa via accelerated apoptosis increases intestinal permeability in experimental severe acute pancreatitis. The Journal of surgical research.

[19] Deng WS (2015). Arpin contributes to bacterial translocation and development of severe acute pancreatitis. World journal of gastroenterology: WJG.

[20] Ahmad R, Rah B, Bastola D, Dhawan P, Singh AB (2017). Obesity-induces Organ and Tissue Specific Tight Junction Restructuring and Barrier Deregulation by Claudin Switching. Scientific reports.

[21] Sukhotnik I (2007). The effect of leptin on intestinal recovery following ischemia-reperfusion injury in a rat. Pediatric surgery international.

[22] Cui H, Lopez M, Rahmouni K (2017). The cellular and molecular bases of leptin and ghrelin resistance in obesity. Nature reviews. Endocrinology.

[23] Drew JE (2007). Insulin, leptin, and adiponectin receptors in colon: regulation relative to differing body adiposity independent of diet and in response to dimethylhydrazine. American journal of physiology. Gastrointestinal and liver physiology.

[24] Barrenetxe J (2002). Distribution of the long leptin receptor isoform in brush border, basolateral membrane, and cytoplasm of enterocytes. Gut.

[25] Gan L (2012). TNF-alpha up-regulates protein level and cell surface expression of the leptin receptor by stimulating its export via a PKC-dependent mechanism. Endocrinology.

[26] Konturek, P. C. *et al*. Leptin modulates the inflammatory response in acute pancreatitis. *Digestion* **65**, 149–160, 64935 (2002). 10.1159/000064935 12138320

[27] Jaworek J (2002). Leptin protects the pancreas from damage induced by caerulein overstimulation by modulating cytokine production. Pancreatology: official journal of the International Association of Pancreatology (IAP)… [et al.].

[28] Matarese G, Lechler RI (2004). Leptin in intestinal inflammation: good and bad gut feelings. Gut.

[29] Turnbaugh PJ (2006). An obesity-associated gut microbiome with increased capacity for energy harvest. Nature.

[30] Geurts L (2011). Altered gut microbiota and endocannabinoid system tone in obese and diabetic leptin-resistant mice: impact on apelin regulation in adipose tissue. Frontiers in microbiology.

[31] Schwab C (2014). Longitudinal study of murine microbiota activity and interactions with the host during acute inflammation and recovery. The ISME journal.

[32] Kaiko GE (2016). The Colonic Crypt Protects Stem. Cells from Microbiota-Derived Metabolites. Cell.

[33] Cho I, Blaser MJ (2012). The human microbiome: at the interface of health and disease. Nature reviews. Genetics.

[34] Beaumont M (2016). Heritable components of the human fecal microbiome are associated with visceral fat. Genome biology.

[35] Del Chierico F (2017). Gut microbiota profiling of pediatric nonalcoholic fatty liver disease and obese patients unveiled by an integrated meta-omics-based approach. Hepatology (Baltimore, Md.).

[36] Pammi M (2017). Intestinal dysbiosis in preterm infants preceding necrotizing enterocolitis: a systematic review and meta-analysis. Microbiome.

[37] Wang, G., Li, X., Zhao, J., Zhang, H. & Chen, W. Lactobacillus casei CCFM419 attenuates type 2 diabetes via a gut microbiota dependent mechanism. *Food & function*, 10.1039/c7fo00593h (2017). 10.1039/c7fo00593h 28782784

[38] Milani C (2016). Gut microbiota composition and Clostridium difficile infection in hospitalized elderly individuals: a metagenomic study. Scientific reports.

[39] Huttunen R, Syrjanen J (2013). Obesity and the risk and outcome of infection. International journal of obesity (2005).

[40] Claesson MJ, Clooney AG, O’Toole PW (2017). A clinician’s guide to microbiome analysis. Nature reviews. Gastroenterology & hepatology.

[41] Hariri N, Thibault L (2010). High-fat diet-induced obesity in animal models. Nutrition research reviews.

[42] Bourgeois F, Alexiu A, Lemonnter D (2007). Dietary-induced obesity: effect of dietary fats on adipose tissue cellularity in mice. British Journal of Nutrition.

[43] Chan YC, Leung PS (2007). Acute pancreatitis: animal models and recent advances in basic research. Pancreas.

[44] Laukkarinen JM, Van Acker GJ, Weiss ER, Steer ML, Perides G (2007). A mouse model of acute biliary pancreatitis induced by retrograde pancreatic duct infusion of Na-taurocholate. Gut.

[45] Schmidt J (1992). A better model of acute pancreatitis for evaluating therapy. Annals of surgery.

[46] Wang X, Wang B, Wu J, Wang G (2001). Beneficial effects of growth hormone on bacterial translocation during the course of acute necrotizing pancreatitis in rats. Pancreas.

[47] Gao JH (2013). Celecoxib ameliorates portal hypertension of the cirrhotic rats through the dual inhibitory effects on the intrahepatic fibrosis and angiogenesis. PloS one.

[48] Caporaso JG (2010). QIIME allows analysis of high-throughput community sequencing data. Nature methods.

[49] Edgar RC, Haas BJ, Clemente JC, Quince C, Knight R (2011). UCHIME improves sensitivity and speed of chimera detection. Bioinformatics.

