# Supplementary material for: Obesity Aggravates Acute Pancreatitis via Damaging Intestinal Mucosal Barrier and Changing Microbiota Composition in Rats
Source: Sci Rep. 2019 Jan 11;9:69. doi: 10.1038/s41598-018-36266-7 (PMC6329748; doi:10.1038/s41598-018-36266-7)
Supplement: Supplementary file 1 — Supplementary information [file 41598_2018_36266_MOESM1_ESM.doc]

| **Obesity Aggravates Acute Pancreatitis via Damaging Intestinal Mucosal Barrier and Changing Microbiota Composition in Rats**  **Cheng Ye ^1^, Ling Liu ^1^, Xiao Ma ^1^, Huan Tong ^1^, Jinhang Gao ^2^, Yang Tai ^1^, Libin Huang ^1^, Chengwei Tang ^1,2*^, Rui Wang ^1*^**  **Supplementary Table 1 Weight and blood fat of the four groups** | | | | |  |
| --- | --- | --- | --- | --- | --- |
| **Variables** | **Groups** | | | |  |
|  | **NC** | **NAP** | **OC** | **OAP** | |
| **Weight (g)** | 301.55±34.95 | 325.50±55.08 | 606.40±106.72 ^a#^ | 610.11±97.37 ^b#^ | |
| **Lee's Index (g1/3×1000/cm)** | 292.46±14.14 | 298.26±16.99 | 316.53±14.46 ^a^**^*^** | 318.16±17.58 ^b^**^*^** | |
| **Cholesterol (mmol/L)** | 1.34±0.34 | 1.17±0.65 | 2.29±0.37 ^a#^ | 2.30±0.42 ^b^**^*^** | |
| **Triglycerides (mmol/L)** | 0.78±0.35 | 0.78±0.35 | 4.23±1.92 ^a#^ | 3.25±1.38 ^b#^ | |
| Note: ^a^ *vs.* NC, ^b^ *vs.* NAP; **^*^** *p*<0.05, ^#^ *p*<0.01 | | | | |  |

| **Supplementary Table 2**  **Primer sequences used in this study for gene expression** | |
| --- | --- |
| **Gene Sequence** | |
| *Leptin* | Forward 5’-CACCCCATTCTGAGTTTGTCC-3’  Reverse 5’-GTTCTCCAGGTCATGAGCTATC-3’ |
| *Ob-R* | Forward 5’-GAATCTAGCCGAGAAGATCCC-3’  Reverse 5’-TTCAGCGTAGCGGTGATG-3’ |
| *Occludin* | Forward 5’-CCCATCTGACTATGCGGAAAG-3’  Reverse 5’-CGGACAAGGTCAGAGGAATC-3’ |
| *Claudin-1* | Forward 5’-CTGGGGACAACATCGTGACT-3’  Reverse 5’-CACAAAGATTGCGATCAGCC-3’ |
| *GAPDH* | Forward 5’-TATGACTCTACCCACGGCAAGT-3’  Reverse 5’-ATACTCAGCACCAGCATCACC-3’ |


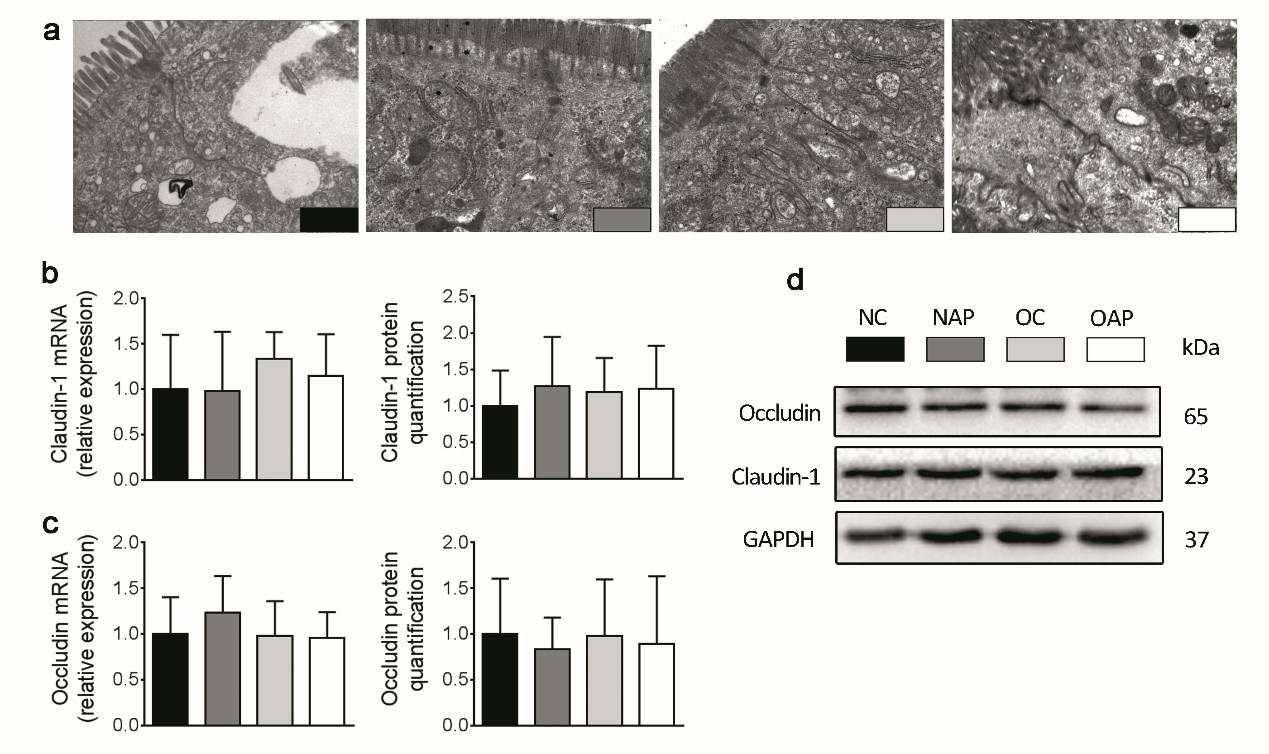


**Supplementary Figure 1 Expressions of tight junction proteins in intestinal epithelial cells**

a: Ultrastructural changes of ileum (transmission electron microscope, ×3,000 magnifications); b: mRNA and protein (Grey value analysis of Western blot by Bio-Rad image lab software 5.1) levels of claudin-1 in ileum; c: mRNA and protein levels of occludin in ileum. d: Western blot analysis of claudin-1 and occludin. Full-length blots are presented in Supplementary Figure 3.

**Supplementary Figure 2: Full length blots for Fig. 2**


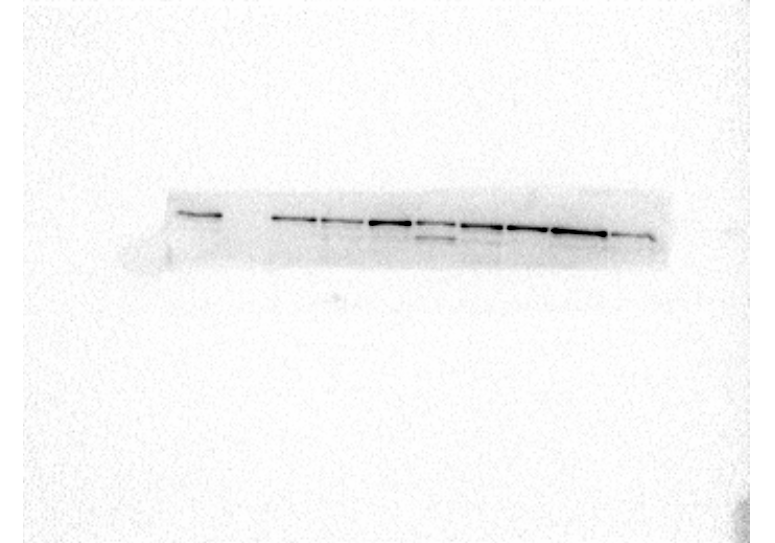
 NC NAP OC OAP NC NAP OC OAP NC NAP OC OAP NC NAP OC OAP


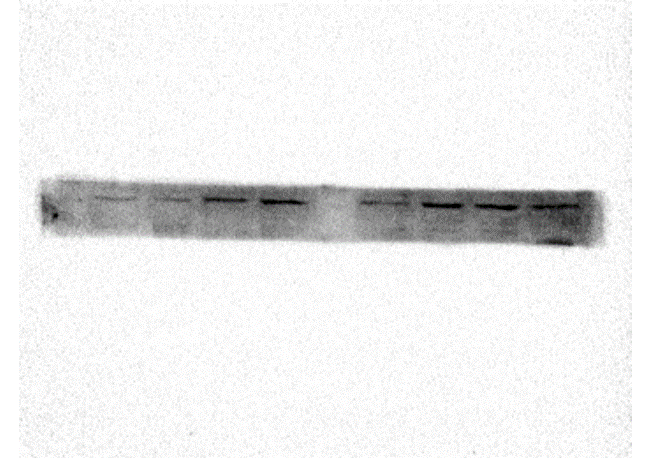

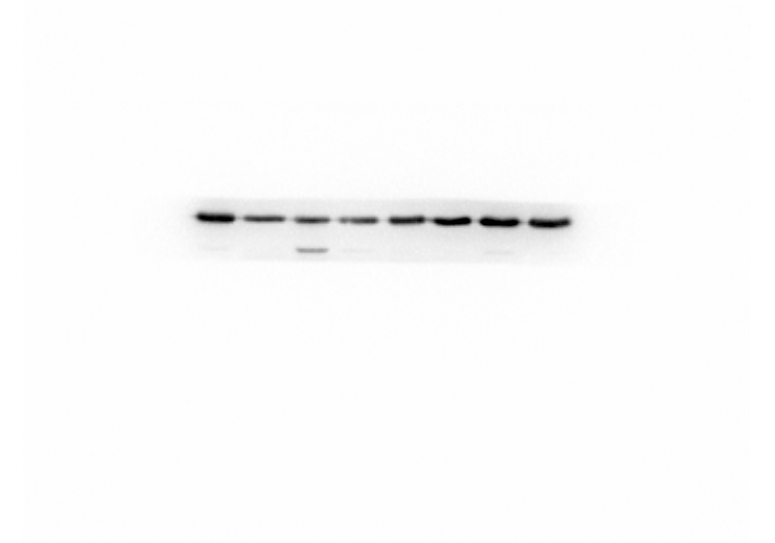

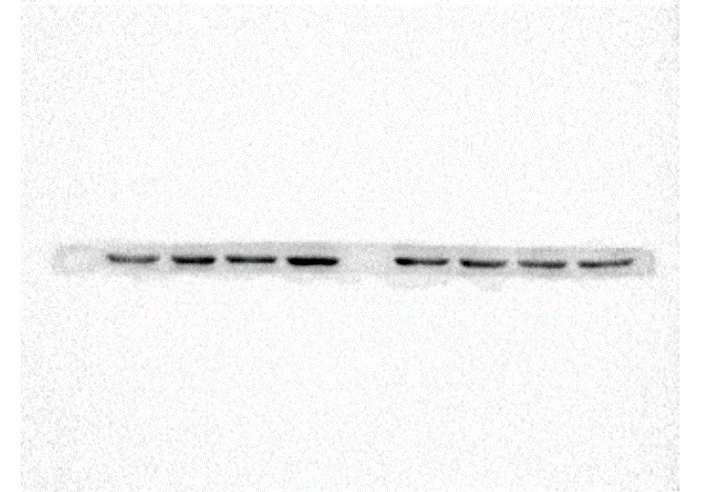


**Leptin Ob-R**

**GAPDH GAPDH**

**Supplementary Figure 3: Full length blots for Supplementary Fig. 1**


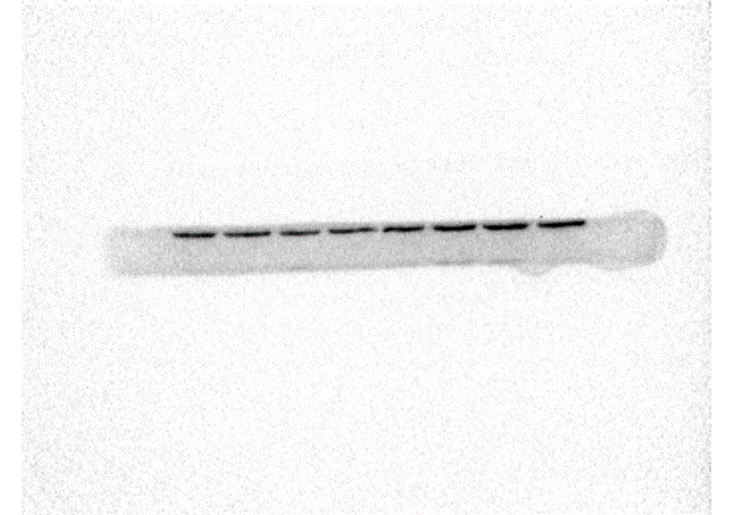

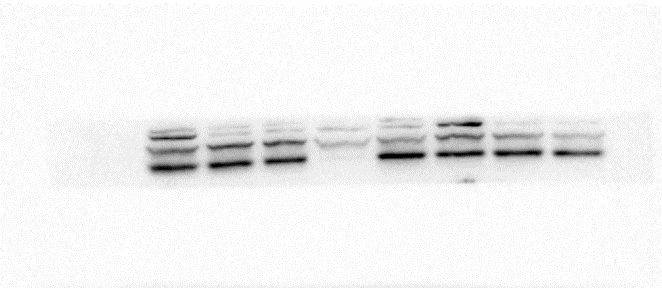

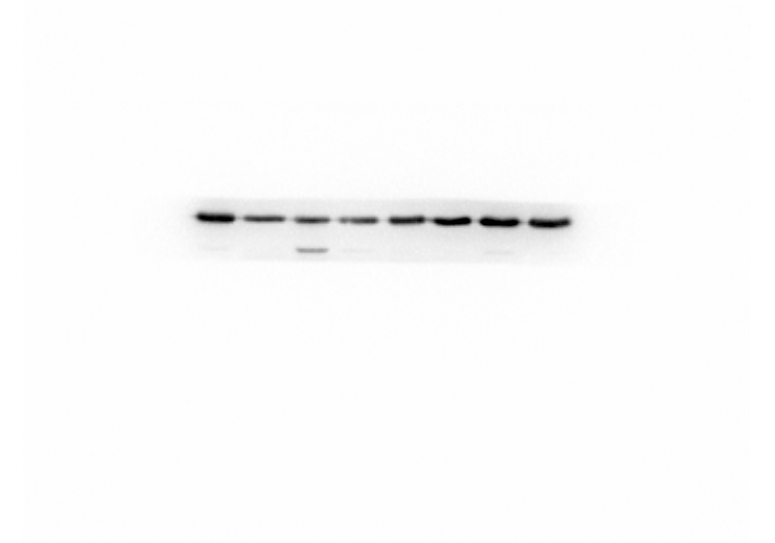
 NC NAP OC OAP NC NAP OC OAP

**Occludin**

**Claudin-1**

**GAPDH**

In order to respond to the reviewers’ questions, we conducted supplementary experiments as followed:

***Reviewer: Faecal samples could be a way to assess variability between groups before treatment.***

Twenty-four hours after AP induction, faecal samples were collected and stored at -80°C (n=8 in each group). DNA extraction, sequencing and statistical analysis were conducted as mentioned in the manuscript for ileal content sample.
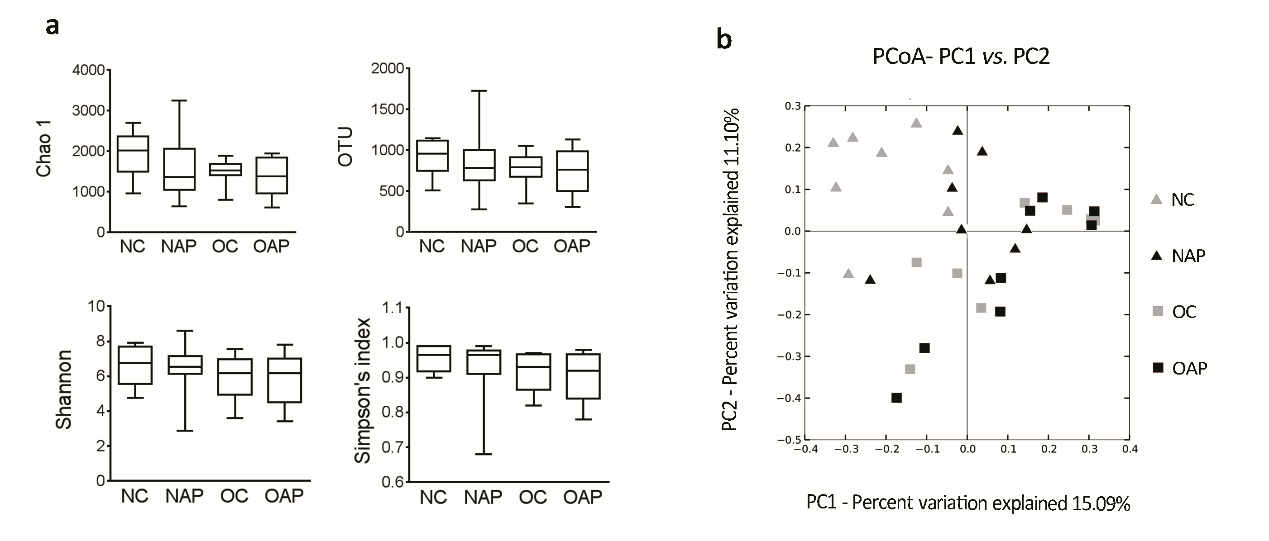


**Supplementary Figure 4 Alpha diversity and beta diversity estimates of the bacterial communities**

a: The Chao 1, OTUs, Shannon and Simpson indexes were used to estimate the richness and diversity of the faecal microbiota, no significant difference was observed between each two groups. b: PCoA based on unweighted Unifrac distance used to reveal the structural segregation of bacterial communities among the four groups. Each value was the mean ± SD from 8 animals in each group and duplicate measurements were made.
